# Global Inequality of Opportunity in Education Decreased During the 20th Century

**DOI:** 10.1111/1468-4446.70105

**Published:** 2026-03-17

**Authors:** Michael Grätz, Mobarak Hossain, Martina Beretta

**Affiliations:** ^1^ Swiss Centre of Expertise in Life Course Research LIVES University of Lausanne Swedish Institute for Social Research SOFI Stockholm University Lausanne Switzerland; ^2^ Department of Social Policy London School of Economics and Political Science London UK; ^3^ Department of Social Policy and Intervention and Nuffield College University of Oxford Oxford UK

**Keywords:** education, equality of opportunity, inequality, life outcomes, world

## Abstract

We document changes in global inequality of opportunity in education for women and men born between 1941 and 1983, using individual‐level census and survey data on 46.7 million individuals from 95 countries, representing all major regions of the world. We measure global inequality of opportunity in education as inequality in education due to circumstances beyond the control of individuals. In addition to gender and social origin, we treat a person's country of residence as a circumstance that produces inequality of opportunity, because the country of residence is, to a large extent, beyond an individual's control. We test whether global inequality of opportunity in education has increased or decreased across cohorts. Our results show a decline in global inequality of opportunity. The decline is stronger among women than men, although inequality of opportunity remains higher among women than men.

## 1 Introduction and Background

A large literature in the social sciences estimates inequality of opportunity in life outcomes such as education, occupation, income, and wealth (Black and Devereux 2011; Breen and Jonsson 2005; Torche 2015). This research measures inequality of opportunity as the effects of the three types of ascribed characteristics (i) social origin, (ii) gender, and (iii) race/ethnicity/migration background on life outcomes. This research is motivated by the normative notion that circumstances beyond the control of individuals should not affect their chances of achieving high levels of education, occupation, income, or wealth (Cohen 2008; Dworkin 2008; Rawls 1971; Roemer 1998). However, almost all of this research has been carried out estimating inequality of opportunity within individual countries, with a strong focus on Europe and the United States.

As a result, the vast majority of research on inequality of opportunity did not estimate global inequality of opportunity because it omitted an important circumstance beyond the control of an individual: the country someone happens to live in, which is often the country in which someone was born. The only exception we are aware of is an empirical study that measured global inequality of opportunity in income, and examined how much inequality in income is explained by country of residence (Milanovic 2015).

We use individual‐level data from 95 countries on 46.7 million women and men born between 1941 and 1983 to study changes across cohorts in global inequality of opportunity in education. We combine data from a previous effort to pool census data across different countries worldwide (Hossain and Beretta 2025) and the European household survey “European Union Statistics on Income and Living Conditions” (EU‐SILC) (Eurostat 2024). We focus on education as a key life outcome because it affects income, work, wealth, health, life expectancy, and well‐being (Lager and Torssander 2012; Lutz and Kc 2011; OECD 2024). We investigate the following research question: Has global inequality of opportunity in education increased or decreased across birth cohorts 1941 to 1983? Answering this research question is important to understand whether the world overall has become more just or unjust in the distribution of education during the 20th century.

Political philosophers debate whether equality of opportunity should be measured at the global level (Tan 2021), or whether the concept of equality of opportunity should be applied only within nation states (Miller 2007). Our study provides an empirical assessment of how much is at stake in this debate. For this purpose, we also compare the contribution of parental education as a measure of social origin and country of residence to global inequality of opportunity. In line with the earlier study on global inequality in income (Milanovic 2015), we only observe the country of residence and not the country of birth. However, migration flows were very low if seen in relation to the total population size for the cohorts we look at (Pritchett 2006).

Our estimates of inequality of opportunity in education should be interpreted as lower bound estimates, as we are not able to measure all circumstances beyond the control of the individual that affect education and as there is certainly measurement error in our data. However, our main contribution, compared to previous research, is to estimate inequality of opportunity using a circumstance, the country of residence, that previous research did not take into account.

## 2 Methods

### 2.1 Data

We use pooled census and survey individual‐level data on 68 countries (Hossain and Beretta 2025) from IPUMS International (University of Minnesota 2025). Only India and Nigeria have survey data from this source. We also employ survey data on 27 countries from the EU‐SILC. These data were collected by national statistical agencies and harmonized by Eurostat to ensure national representativeness (Eurostat 2024). Our total sample includes 95 countries and 46,689,677 individuals born between 1937 and 1988, drawn from 294 censuses and surveys conducted between 1960 and 2017. Table 1 gives an overview of the countries and observations. We limit the age range at which education is measured to 25–60 for high‐income countries (HICs) and 21–60 for low‐ and middle‐income countries (LMICs) (Hossain and Beretta 2025), as average schooling levels tend to be lower and completed earlier in the latter group. Recent research on educational inequality using similar data shows that the inequality measures are not sensitive to selecting higher age ranges (Hossain and Beretta 2025). Consequently, our analysis using a more conservative age range of 25–60 for LMICs yields virtually identical estimates, as shown in Supporting Information S1: Figure S1.

**TABLE 1:** Number of observations per country.

Country	Observations	Country	Observations	Country	Observations	Country	Observations
Argentina	1,008,582	Estonia	10,431	Latvia	10,841	Portugal	11,456
Armenia	85,424	Ethiopia	344,722	Lesotho	38,691	Puerto Rico	59,518
Austria	12,726	Fiji	38,162	Lithuania	10,879	Russia	1,346,361
Bangladesh	1,647,975	Finland	11,911	Luxembourg	11,984	Rwanda	102,738
Belarus	144,175	France	22,385	Malawi	99,381	Senegal	310,375
Belgium	11,452	Germany	25,543	Malaysia	95,131	Sierra Leone	77,653
Benin	119,265	Ghana	316,521	Mali	163,941	Slovak Republic	15,176
Bolivia	198,994	Greece	13,009	Mauritius	50,053	Slovenia	9932
Botswana	45,830	Guatemala	194,673	Mexico	4,162,037	South Africa	1,711,435
Brazil	9,209,320	Guinea	131,618	Mongolia	32,852	Spain	33,303
Burkina Faso	89,789	Haiti	96,252	Morocco	1,102,781	Sweden	6220
Cambodia	215,715	Honduras	91,094	Mozambique	124,721	Tanzania	483,951
Cameroon	153,228	Hungary	22,768	Nepal	465,052	Thailand	211,948
Chile	483,595	Iceland	3508	Netherlands	11,334	Togo	19,297
China	3,283,914	India	361,443	Nicaragua	100,347	Trinidad and Tobago	46,884
Colombia	1,103,171	Indonesia	4,715,973	Nigeria	22,562	Turkey	683,922
Costa Rica	129,067	Iran	345,822	Norway	6486	Uganda	286,078
Cuba	302,137	Ireland	7946	Palestine	111,284	United Kingdom	15,461
Cyprus	10,558	Italy	49,916	Panama	114,257	United States	1,645,087
Czech Republic	12,282	Jamaica	50,601	Papua New Guinea	49,043	Uruguay	145,112
Denmark	6488	Jordan	62,496	Paraguay	116,403	Venezuela	59,9574
Dominican Republic	215,043	Kenya	346,591	Peru	504,179	Vietnam	2,110,304
Ecuador	408,914	Kyrgyz Republic	73,790	Philippines	2,883,666	Zambia	150,414
El Salvador	853	Laos	46,335	Poland	37,566		

### 2.2 Variables

We use years of education as our outcome variable. For a small number of countries where years of education were not reported, we converted levels of educational attainment into years of education using UNESCO's International Standard Classification of Education (ISCED). We measure social origin using the highest value of maternal or paternal years of education (Erikson 1984). In cases in which education is only available for one parent, we use this value. We employ a dummy variable for gender.

Since we account for parental years of education in our analysis, it is important to verify that our sample is not biased by differences in household composition between respondents who live and those who do not live with their parents. Supporting Information S1: Figure S2 compares the average years of education between these groups. The strong correlation (*r* = 0.97) between the two groups suggests that the educational outcomes are very similar regardless of cohabitation status. This comparison is particularly relevant for LMICs using census data, where the household structure is often not fully observed. The similarity in outcomes indicates that selection bias due to using household census data is low, consistent with previous research on intergenerational mobility (Hossain and Beretta 2025).

### 2.3 Analytical Strategy

Our analytical approach follows a literature that has been used to measure inequality of opportunity within countries (Brunori 2016; Checchi and Peragine 2009; Ferreira and Gignoux 2011; Hufe et al. 2017). This approach uses the mean log deviation (MLD) to estimate inequality in an outcome (in our case, education).^1^ A counterfactual distribution of education is then calculated by estimating a regression model with education as the outcome and circumstances as the predictor variables. In our case, the circumstances are the country of residence, parental education, and gender. The ratio of the MLD of the counterfactual distribution compared to the MLD of the actual distribution is the measure of inequality of opportunity. It is the share of education that is explained by the circumstances. We use Stata's ineqdeco package to estimate the MLD ratio (Jenkins 1999).

As an alternative metric, we report in Supporting Information S1: Figure S4 the results using the explained variance (*R* ^2^) of a regression model that includes our measures of circumstances as independent variables to assess inequality of opportunity. This alternative approach has also been used in previous research on inequality of opportunity (Björklund and Jäntti 2020; Ferreira and Gignoux 2014). The results using *R* ^2^ are fully in line with those obtained using the MLD ratio.

**FIGURE 1:**
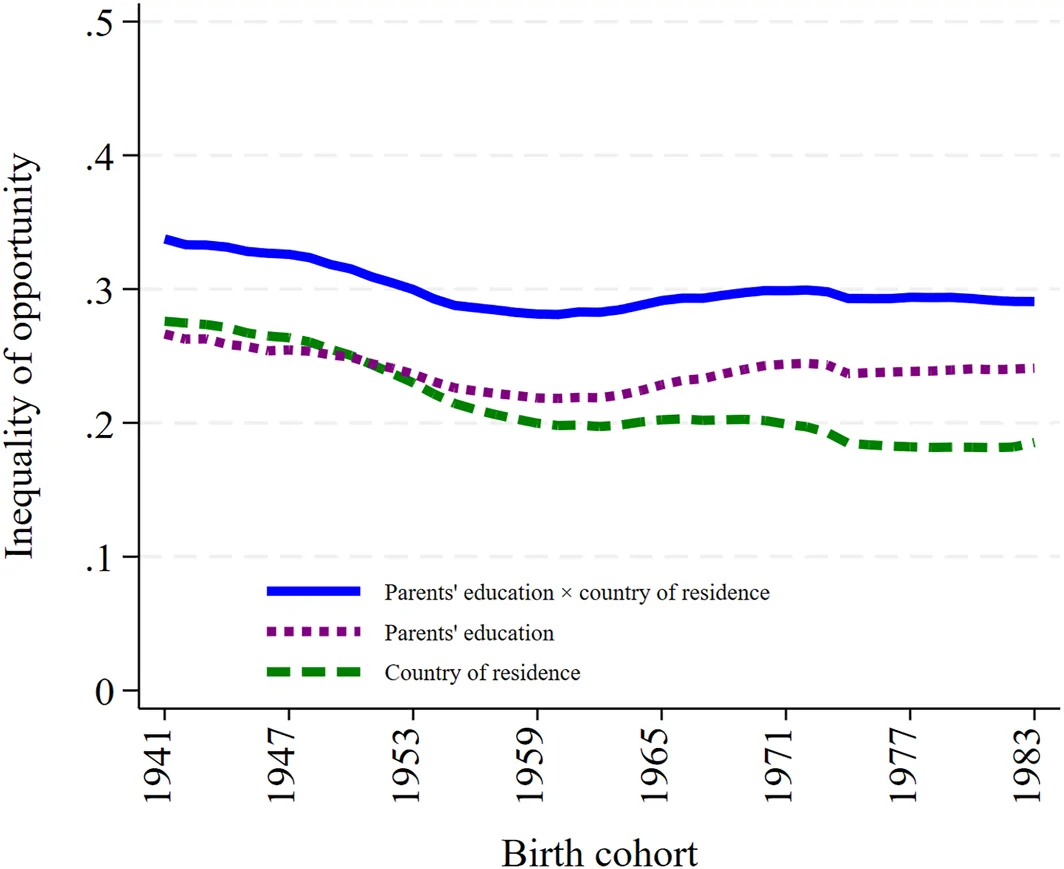
Changes in global inequality of opportunity in education for birth cohorts 1941–1983.

We estimate the models separately for each cohort born between 1941 and 1983. The outcome of the models is education; the independent variables are: country of residence, the highest level of parental education, and gender. We estimate three models: one that includes country of residence via 94 dummy variables; one that includes parental years of education; and one that includes country of residence via the 94 dummy variables, parental years of education, and the interactions among these variables. All models include gender. We also control for the census or survey year in all models. In Figure 1 below, we report the ratio of the MLD in education explained by each of these models.

For each year from 1941 to 1983, we define a cohort by pooling individuals born in that year, the four years before, and the five years after. For example, the 1941 birth cohort includes all individuals born from 1937 to 1946. This approach increases cohort sample sizes, smooths year‐to‐year fluctuations, and addresses occasional missing data in specific birth cohort‐country combinations. We have 43 overlapping birth cohorts, the final one in 1983, including individuals born up to 1988.

All analyses are weighted by country–cohort population size so that each country's contribution reflects the size of its population in a given birth cohort.^2^ We do not disaggregate results by region because this would control away the effect of between‐region differences on global inequality of opportunity that we are interested in studying.

## 3 Results

### 3.1 Global Inequality of Opportunity Has Declined Between Birth Cohorts 1941 and 1983

Figure 1 shows that global inequality of opportunity in education declined between birth cohorts 1941 and 1983.^3^ For the 1941 birth cohort, country of residence and parental education (based on the highest educated parent) and their interaction together explained 34% of the MLD in years of education. The explained MLD falls to 29% for the 1983 cohort. When estimated separately, the MLD for parental education declines from 27 to 24%, while the contribution of the country context drops more sharply from 28 to 19%. The fact that the “joint” inequality of opportunity is lower than the sum of the separate components reflects shared explanatory power; the effect of parental education and country of residence is correlated, as parents in higher‐income countries tend to have more schooling. The steepest declines in global inequality of opportunity occurred for individuals born between the mid‐1940s and late 1950s, with a more gradual or flat pattern in later birth cohorts. Notably, while the role of country of residence declines almost continuously, the explanatory power of parental education stabilizes for those born after the mid‐1960s. These patterns suggest that the global reduction in inequality of opportunity in education has been driven mainly by the reduction in cross‐country differences.

### 3.2 Global Inequality of Opportunity Has Declined More for Women Than Men

**FIGURE 2:**
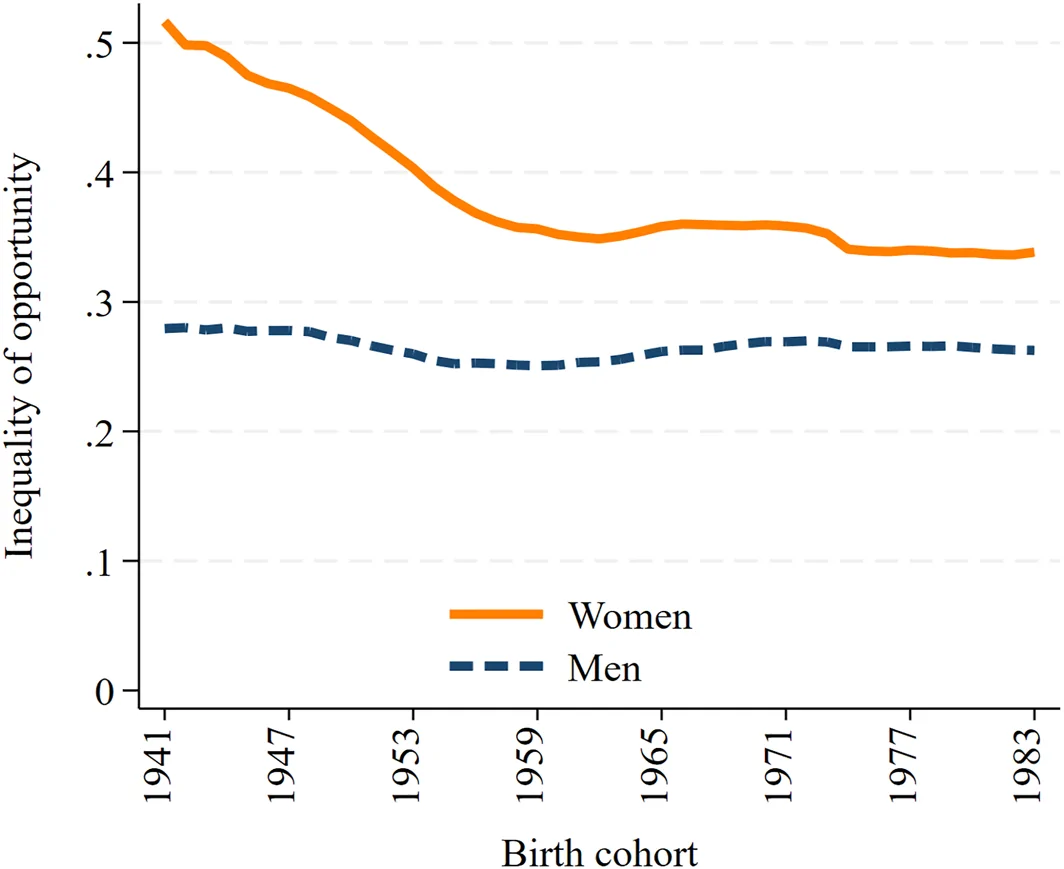
Gender differences in changes in global inequality of opportunity for birth cohorts 1941–1983.

Figure 2 estimates global inequality of opportunity in education separately for women and men.^4^ A decline in global inequality of opportunity is found for both women and men but there are gender differences. In every cohort, inequality of opportunity is higher among women than among men, indicating that the education of women is more strongly affected by circumstances beyond their control. Gender differences are particularly marked in the earlier cohorts. For the 1941 cohort, inequality of opportunity is 52% for women and 28% for men, a difference of 24% points. Both groups experienced declines over time, but the reduction in inequality of opportunity in education is much more pronounced for women than for men. By the 1983 cohort, inequality of opportunity falls to 34% for women and 26% for men, narrowing the gender difference in inequality of opportunity in education to eight percentage points. Despite this convergence, gender differences remain substantial across cohorts. These patterns suggest that, while inequality of opportunity in education has decreased much more sharply for women than men, differences in inequality of opportunity in education persist between men and women.

### 3.3 Global Inequality of Opportunity Has Declined for Primary and Secondary But Not for Tertiary Education

**FIGURE 3:**
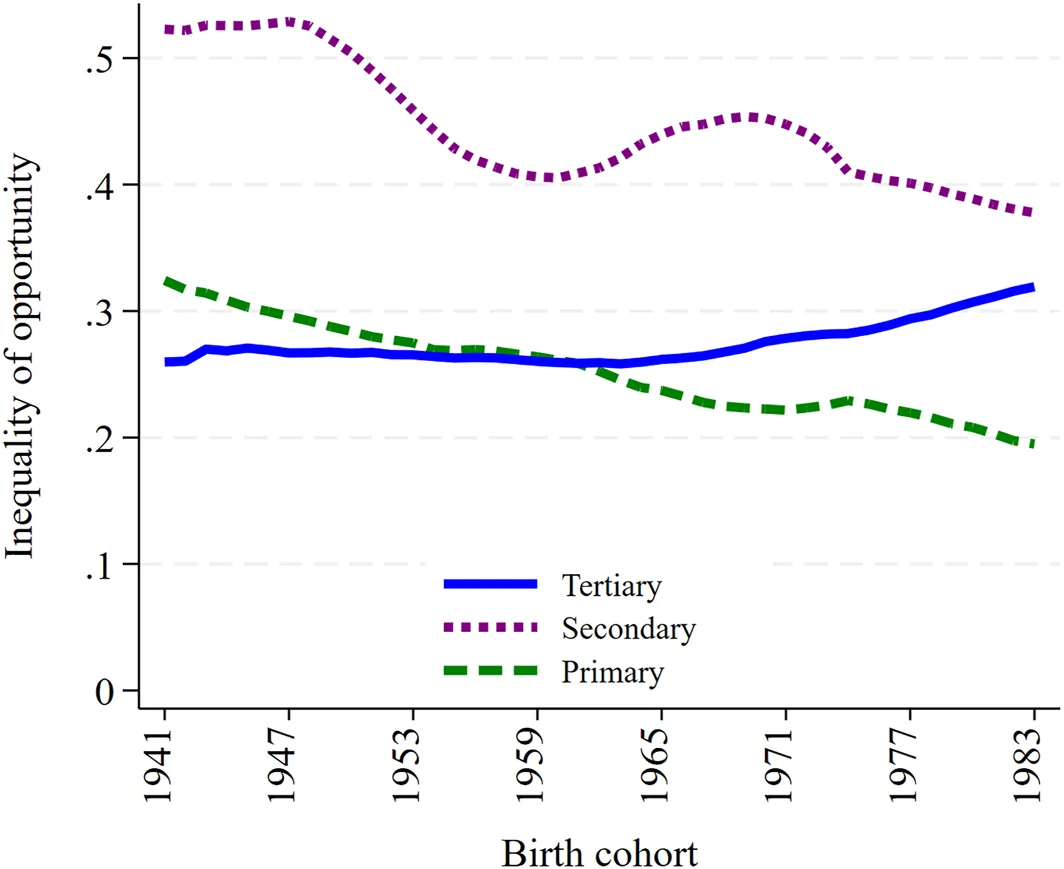
Changes in global inequality of opportunity in education by level of education for birth cohorts 1941–1983.

Figure 3 illustrates changes in global inequality of opportunity in education by level of education.^5^ For this analysis, we recoded educational attainment in several dummy variables based on the highest completed level of education. Primary is a dummy variable, which is coded 1 if a respondent obtained at least a primary education. Secondary is coded 1 for all respondents who obtained at least a secondary education. Consequently, tertiary is coded 1 for respondents who obtained a tertiary education and 0 for those who did not obtain a tertiary education.

The findings show that the decline in global inequality of opportunity is driven by changes in primary and in secondary education. For both these outcomes, we observe a general decline in global inequality of opportunity across cohorts. Global inequality of opportunity in tertiary education has, however, increased across cohorts. The reason for this increase is likely to be the strong expansion of tertiary education in high‐income countries.

### 3.4 Robustness Checks

Supporting Information S1: Figure S4 reports a robustness check that implements an alternative measure of inequality of opportunity: the *R* ^2^. Following Björklund and Jäntti (2020) and Ferreira and Gignoux (2014), we consider *R* ^2^ for its intuitive interpretation as the share of explained variance as a measure of inequality of opportunity. The results in Supporting Information S1: Figure S4 show a decline in global inequality of opportunity, consistent with our findings based on the MLD ratio.

## 4 Discussion

Estimating global inequality of opportunity requires taking the country of residence into account as a circumstance beyond an individual's control (Milanovic 2015). We estimate global inequality of opportunity in education using individual‐level census and survey data from 95 countries. Our analysis shows that global inequality of opportunity in education has decreased across birth cohorts from 1941 to 1983. This decrease is mainly due to a reduction in the role of country of residence in affecting inequality of opportunity in education. The decline in inequality of opportunity in education has been stronger among women than men. However, in all birth cohorts that we analyze the global inequality of opportunity in education is larger among women than men.

We interpret this finding as evidence that the overall decline in global inequality of opportunity in education has been driven largely by opportunities such as greater access to schooling during the 20th century, rather than by a reduction in inequality linked to individual‐level factors alone. This distinction matters. It implies that progress has not come solely from narrowing opportunity gaps within countries, but also from broader systemic shifts that expanded educational access at a global scale. We think here in the first place about educational expansion, which has long been argued to be a main driver behind increasing equality of opportunity (Breen 2010; Pfeffer and Hertel 2015; van de Werfhorst 2024).

Our results show that the world overall has become more just in the distribution of education between birth cohorts 1941 and 1983. However, global inequality of opportunity in education is still very high. Around 29% of inequality in education is due to factors beyond the individual's control, even for the most recent birth cohort included in our data. As said above, this estimate underestimates true inequality of opportunity because of other factors beyond the control of the individual affecting education that we cannot observe in our data.

We also find that the decline in global inequality of opportunity is driven by changes in primary and secondary education. Global inequality of opportunity has steadily decreased across cohorts at these two levels, whereas global inequality in tertiary education shows an increase. These patterns are consistent with evidence from other settings. Blossfeld et al. (2015) showed that long‐term educational expansion in Germany reduced origin‐based inequalities mainly at the early transitions from primary to secondary school, while inequalities at the tertiary level increased slightly. Erikson (2020) argued that performance and choice in the early stages of schooling could influence inequality of opportunity, consistent with the view that expanding access at the foundational levels of education has greater equalizing potential. Evidence from LMICs also points to the importance of targeted expansion. For example, programs that expanded basic education and focused on girls' schooling through scholarships or cash transfers have been shown to have gradually improved access and reduced educational disparities, with sustained positive effects on their children as well (Khandker et al. 2021; Devi et al. 2025; Wu 2022).

While we do not directly observe the mechanisms behind the decline in global inequality of opportunity, the consistency and scale of the decline point to structural forces that extend beyond the family, raising important questions about the role of state policies in influencing educational opportunities. We argue that, in particular, the expansion of primary and secondary education in LMICs (Hossain and Beretta 2025) reduces global inequality of opportunity. Further reductions in global inequality of opportunity are likely to be achieved by the expansion of tertiary education, via policies promoting tertiary education, in these contexts.

Our analysis is limited by the structure of the available data. We observe only final educational attainment and cannot model transitions across schooling levels, which restricts our ability to identify the educational stages at which inequalities emerge or change. Years of schooling also do not capture qualitative dimensions such as curricula, tracking, or school resources, which have been shown to matter for educational inequalities (Breen and Jonsson 2005). These constraints also apply to our analysis of gender differences. While we document higher global inequality of opportunity for women than men, we cannot account for gender differences in educational quality, field of study, or other mechanisms that influence educational trajectories. In addition, global expansions in schooling affect the distribution of educational attainment, which complicates cross‐cohort comparisons.

At the same time, the breadth of our dataset allows us to document global patterns, including for many LMICs, where evidence on educational opportunity is scarce due to limited data. Our approach captures country of residence as a circumstance, which extends beyond what most previous work has been able to consider. Future research using data that track individual transitions or include richer measures of school quality will be important to understand the mechanisms behind the trends we describe, particularly in LMICs, where such evidence remains limited.

## Funding

This work was supported by the Schweizerischer Nationalfonds zur wissenschaftlichen Forschung/Swiss National Science Foundation (SNSF) under grant agreement TMSGI1_211627 and by the Riksbankens Jubileumsfond, Grant No. P24‐0170.

## Conflicts of Interest

The authors declare no conflicts of interest.

## Supporting information


Supporting Information S1

## Data Availability

Data sharing not applicable to this article as no datasets were generated during the current study. All data is publicly available. We have made the code to replicate our analyses available at https://doi.org/10.17605/OSF.IO/X8SUT.

## Endnotes

^1^
Since many individuals present 0 years of education, which would not be defined when log‐transforming, we add 1 to the values of all respondents before conducting the log‐transformation.

^2^
This is to ensure that we truly measure global inequality of opportunity. A disadvantage of this approach is, however, that we cannot obtain standard errors and confidence intervals, which we need to obtain via bootstrap. We can only do so for unweighted estimates. However, given our large sample size, confidence intervals are very small and virtually invisible in the figures. We report the unweighted estimates, including confidence intervals, in Supporting Information S1: Figure S3 and Table S3. They fully confirm the finding of a decline in global inequality of opportunity across cohorts.

^3^
All estimates underlying the figure are reported in Supporting Information S1: Table S1.

^4^
All estimates underlying the figure are reported in Supporting Information S1: Table S1.

^5^
All estimates underlying the figure are reported in Supporting Information S1: Table S2.
