# Supplementary material for: Global Inequality of Opportunity in Education Decreased During the 20th Century
Source: Br J Sociol. 2026 Mar 17;77(4):844–50. doi: 10.1111/1468-4446.70105 (PMC13536524; doi:10.1111/1468-4446.70105)
Supplement: Supplementary file 1 [file BJOS-77-844-s001.pdf]

Online Supplement to Global Inequality of Opportunity in Education Decreased During the 20th Century

February 10, 2026

FIGURES

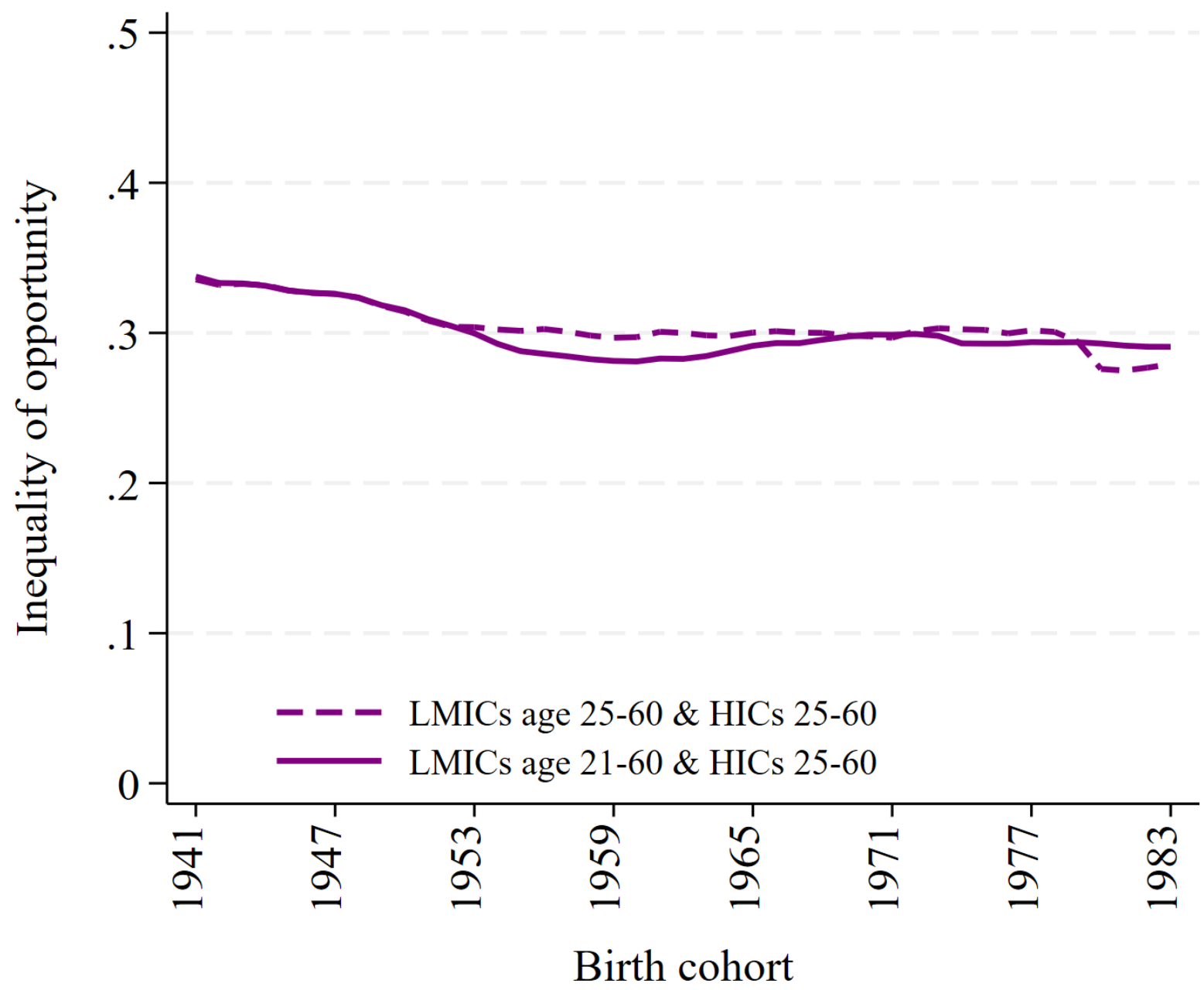

Figure S1: Changes in global inequality of opportunity in education across birth cohorts, measured using Mean Log Deviation (MLD) ratio. The solid line shows the main specifi-cation (LMICs 21—60 and HICs 25—60), while the dashed line shows a robustness check using the same age range (25—60) for LMICs and HICs.

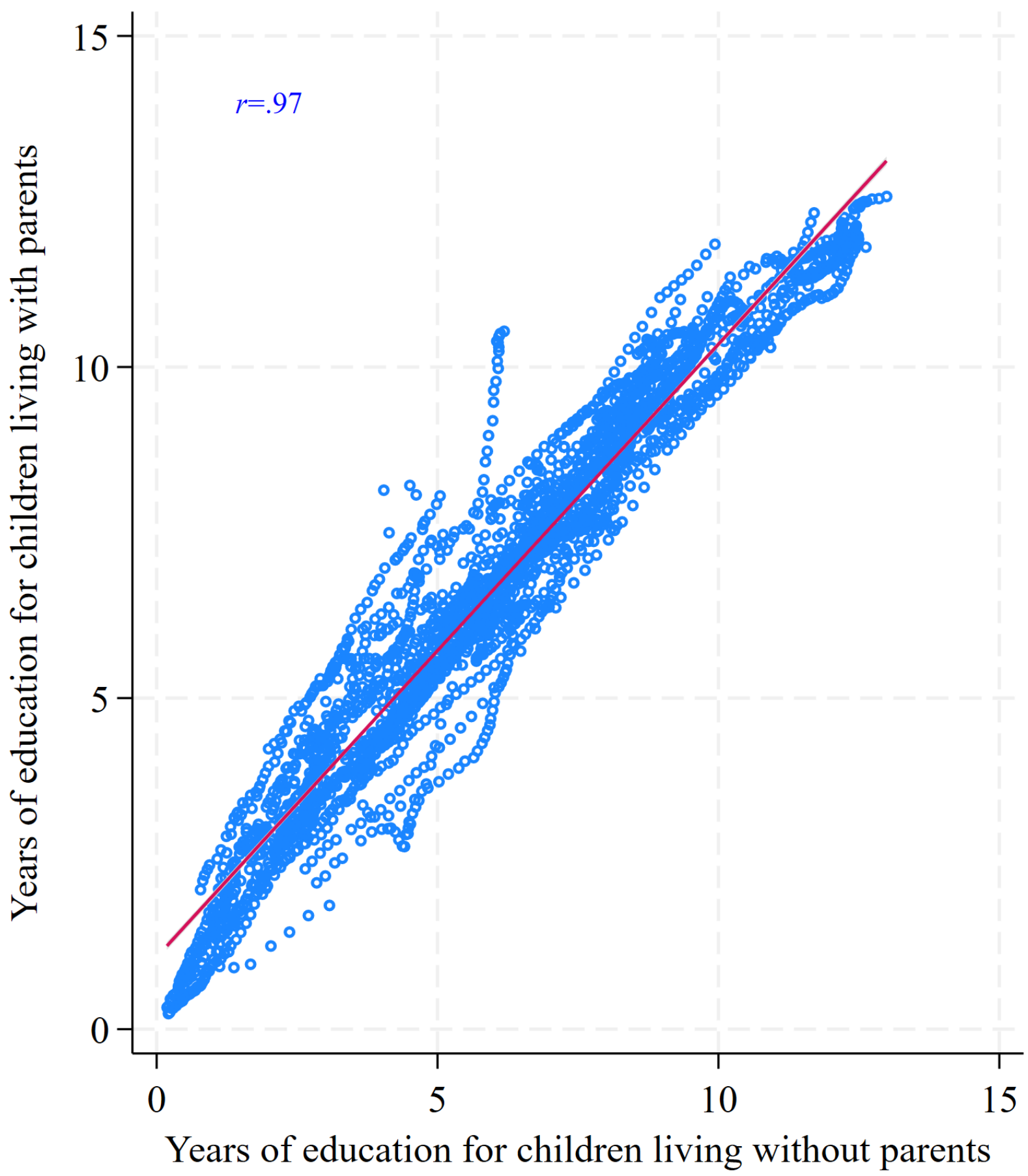

Figure S2: The correlations between years of education for children living with and without parents for each country-birth cohort-combination (IPUMS International).

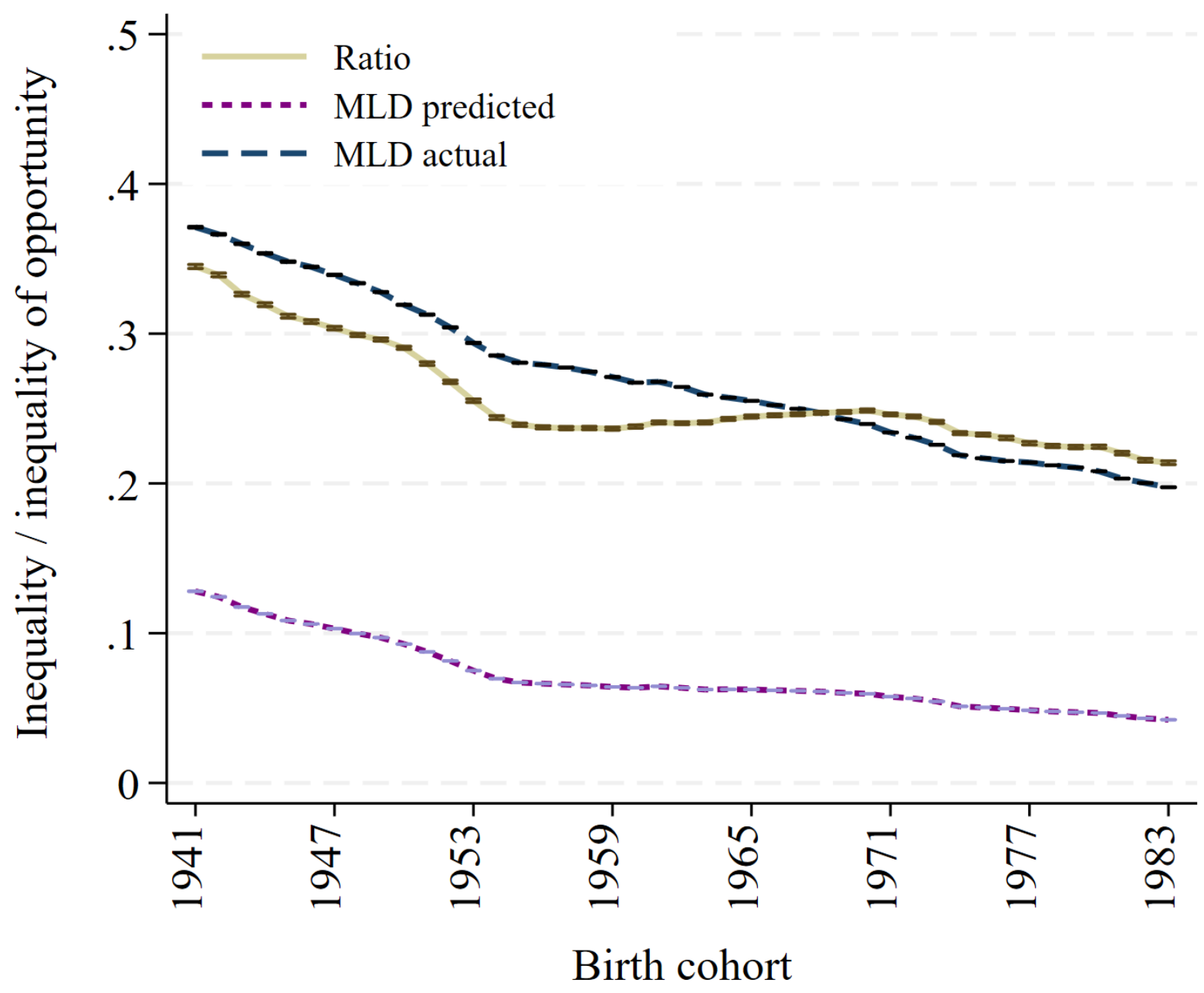

Figure S3: Evolution of inequality in education with 95% confidence intervals (CIs), based on the actual MLD, the MLD predicted by circumstances, and the ratio between predicted and actual MLD. CIs are estimated using bootstrapping using Stata's ineqdeco package (Jenkins, 1999). Because the bootstrap procedure does not allow the use of sampling (and population size) weights, all estimates in this figure are unweighted and therefore differ from the weighted estimates reported in Figure 1. All estimates are reported in Table S3 in the Online Supplement.

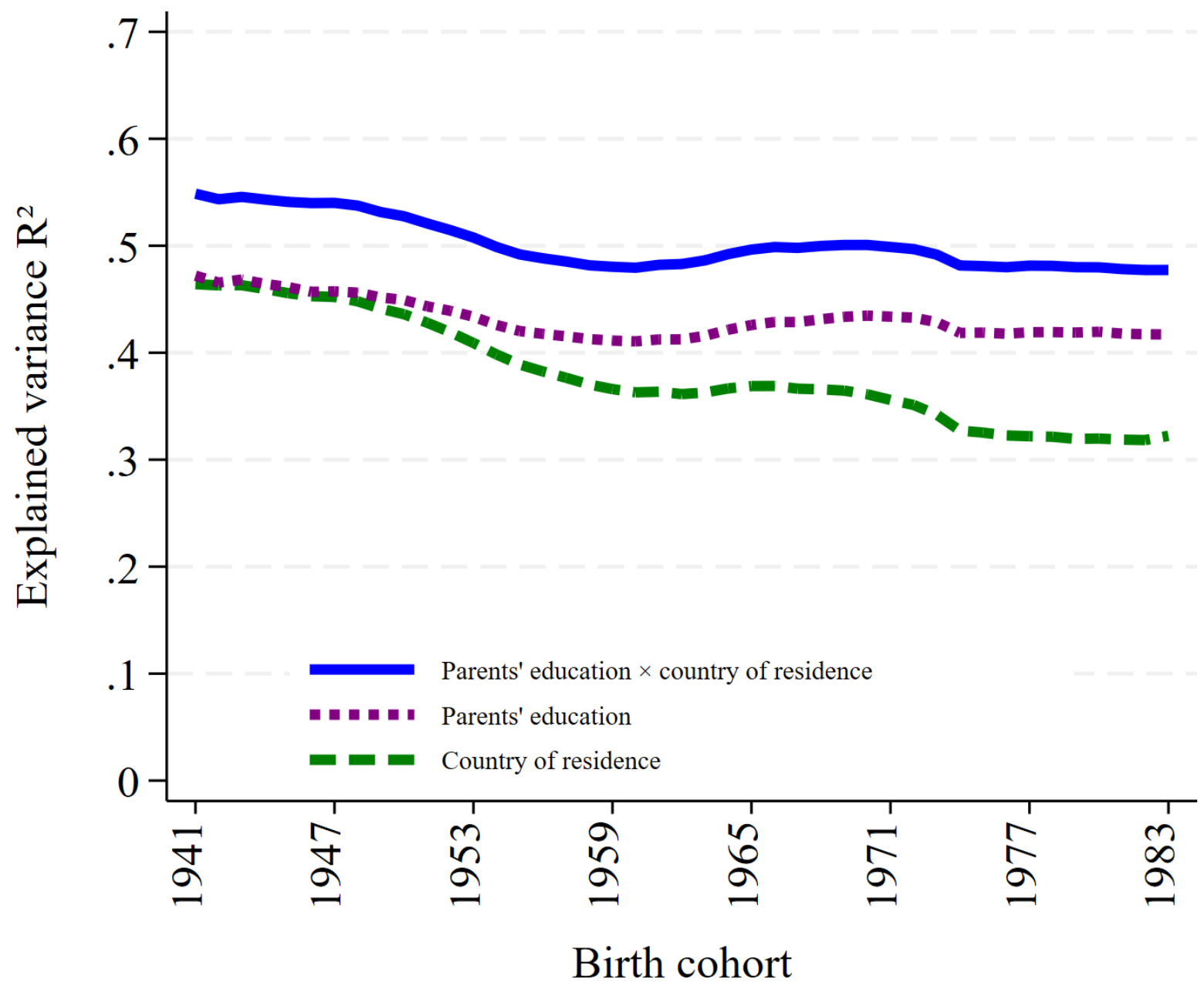

Figure S4: Changes in global inequality of opportunity in education for birth cohorts 1941 to 1983 based on R^2.

TABLES

Table S1: MLD ratios from different models by cohort (Figures 1 and 2)

**cohort**	**mld_ratio_country**	**mld_ratio_parents**	**mld_ratio_cntANDparents**	**mld_men_cntANDparent**	**mld_women_cntANDparent**
1941	0.27597159	0.26646872	0.33752082	0.27939343	0.51643936
1942	0.27469734	0.26262100	0.33325700	0.28008238	0.49834380
1943	0.27355592	0.26283530	0.33295900	0.27833773	0.49778365
1944	0.27130377	0.25871888	0.33144508	0.27992445	0.48906668
1945	0.26719816	0.25706275	0.32813610	0.27728084	0.47500587
1946	0.26490945	0.25394538	0.32679743	0.27781712	0.46849619
1947	0.26371268	0.25450707	0.32604287	0.27792078	0.46501643
1948	0.26051162	0.25356624	0.32356177	0.27699435	0.45843690
1949	0.25514795	0.25085913	0.31845528	0.27252901	0.44933914
1950	0.25040328	0.24922219	0.31509755	0.27016368	0.43989286
1951	0.24355464	0.24426103	0.30916728	0.26606513	0.42730823
1952	0.23691677	0.24087632	0.30465415	0.26269073	0.41557093
1953	0.22999951	0.23652531	0.29973570	0.25985688	0.40345080
1954	0.22163930	0.23076810	0.29273064	0.25474303	0.38903939
1955	0.21451339	0.22621875	0.28784160	0.25223906	0.37787922
1956	0.20998536	0.22388059	0.28608779	0.25282887	0.36870573
1957	0.20617107	0.22197334	0.28442841	0.25227826	0.36213496
1958	0.20279929	0.22039070	0.28251235	0.25120147	0.35755896
1959	0.19972484	0.21859497	0.28135304	0.25070511	0.35632699
1960	0.19806489	0.21821057	0.28098549	0.25109427	0.35210313
1961	0.19839914	0.21907426	0.28294092	0.25326651	0.35004526
1962	0.19723682	0.21874252	0.28270829	0.25374773	0.34866446
1963	0.19839884	0.22085984	0.28460187	0.25558434	0.35082604
1964	0.20072407	0.22418374	0.28801648	0.25876875	0.35427020
1965	0.20231572	0.22848181	0.29141219	0.26172529	0.35828745
1966	0.20302386	0.23175383	0.29324464	0.26272903	0.36009262
1967	0.20184490	0.23307047	0.29318620	0.26277046	0.35966231
1968	0.20232396	0.23677735	0.29547602	0.26562334	0.35919838
1969	0.20268409	0.23996757	0.29738684	0.26767411	0.35877337
1970	0.20171471	0.24290498	0.29890273	0.26930571	0.35951438
1971	0.19913156	0.24397533	0.29875531	0.26923008	0.35856167
1972	0.19706981	0.24454210	0.29932639	0.26990117	0.35695169
1973	0.19284143	0.24335472	0.29804898	0.26902689	0.35267875
1974	0.18475585	0.23678832	0.29300834	0.26536354	0.34072393
1975	0.18360438	0.23752673	0.29287287	0.26528484	0.33928174
1976	0.18269284	0.23794992	0.29287515	0.26538624	0.33881895
1977	0.18207761	0.23843919	0.29388014	0.26588745	0.34000667
1978	0.18162990	0.23878530	0.29370173	0.26559627	0.33930956
1979	0.18194331	0.23955612	0.29384170	0.26609612	0.33777628
1980	0.18177139	0.24037805	0.29292638	0.26484258	0.33807688
1981	0.18148731	0.23978338	0.29155589	0.26372842	0.33666909
1982	0.18199346	0.24021972	0.29079535	0.26296791	0.33630555
1983	0.18531967	0.24089896	0.29073843	0.26254973	0.33842797

Table S2: MLD ratios by education level and cohort (Figure 3)

**cohort**	**mld_cntANDparent_primary**	**mld_cntANDparent_secondary**	**mld_cntANDparent_tertiary**
1941	0.32423212	0.52281021	0.25976041
1942	0.31700961	0.52188459	0.26038320
1943	0.31420066	0.52595218	0.26986199
1944	0.30851348	0.52568605	0.26853776
1945	0.30310573	0.52552386	0.27070453
1946	0.29971246	0.52713396	0.26906498
1947	0.29589849	0.52889938	0.26690982
1948	0.29211445	0.52535738	0.26701912
1949	0.28774415	0.51496762	0.26764271
1950	0.28396461	0.50430016	0.26660522
1951	0.27976821	0.49004703	0.26732256
1952	0.27715341	0.47476049	0.26546288
1953	0.27477104	0.45854842	0.26537503
1954	0.26932899	0.44277863	0.26405789
1955	0.26876181	0.42850171	0.26284157
1956	0.26957083	0.41958977	0.26327747
1957	0.26834564	0.41364564	0.26291395
1958	0.26598789	0.40846340	0.26157500
1959	0.26382502	0.40607474	0.26022069
1960	0.26105400	0.40527912	0.25924803
1961	0.25842953	0.40913527	0.25877426
1962	0.25227401	0.41329008	0.25920695
1963	0.24544826	0.42110575	0.25821691
1964	0.23959534	0.43201529	0.25959964
1965	0.23726237	0.43962062	0.26171082
1966	0.23267395	0.44574962	0.26287448
1967	0.22780652	0.44759726	0.26452108
1968	0.22479255	0.45181069	0.26757453
1969	0.22342240	0.45374014	0.27061628
1970	0.22254922	0.45249778	0.27584871
1971	0.22162800	0.44761736	0.27845219
1972	0.22340444	0.44055720	0.28047291
1973	0.22558009	0.42935593	0.28178142
1974	0.22919122	0.41001057	0.28223753
1975	0.22651971	0.40641905	0.28491770
1976	0.22243732	0.40318937	0.28886568
1977	0.21960329	0.40113327	0.29387795
1978	0.21582562	0.39746652	0.29704515
1979	0.21093066	0.39258561	0.30234469
1980	0.20807180	0.38883403	0.30698836
1981	0.20314341	0.38436533	0.31110265
1982	0.19753166	0.38057154	0.31573297
1983	0.19485109	0.37768704	0.31920445

Table S3: MLD actual, predicted, and ratio with confidence intervals by cohort (Figure S3)

cohort	coef_mld	ci_low_mld	ci_high_mld	coef_mld_predicted	ci_low_mld_predicted	ci_high_mld_predicted	ratio	ci_low_ratio	ci_high_ratio
1941	0.37107277	0.37058006	0.37156549	0.12796384	0.12778711	0.12814057	0.3448484	0.3435130	0.3461889
1942	0.36633954	0.36587172	0.36680735	0.12426071	0.12407943	0.12444199	0.3391955	0.3379099	0.3404859
1943	0.35996122	0.35953935	0.36038309	0.11747179	0.11730951	0.11763407	0.3263457	0.3251666	0.3275290
1944	0.35356911	0.35313459	0.35400363	0.11290851	0.11277231	0.11304471	0.3193393	0.3181066	0.3205768
1945	0.34806399	0.34763707	0.34849091	0.10847624	0.10835293	0.10859955	0.3116560	0.3104269	0.3128900
1946	0.34456496	0.34415209	0.34497783	0.10613861	0.10601638	0.10626085	0.3080366	0.3068357	0.3092422
1947	0.33925368	0.33886708	0.33964028	0.10301481	0.10290285	0.10312677	0.3036513	0.3025095	0.3047974
1948	0.33367742	0.33333180	0.33402305	0.09981171	0.09970804	0.09991538	0.2991264	0.2980882	0.3001681
1949	0.32773103	0.32739681	0.32806526	0.09702608	0.09692028	0.09713187	0.2960540	0.2950314	0.2970801
1950	0.31926009	0.31893982	0.31958035	0.09275917	0.09267146	0.09284689	0.2905442	0.2895397	0.2915523
1951	0.31270020	0.31235031	0.31305009	0.08755444	0.08745902	0.08764986	0.2799948	0.2788748	0.2811193
1952	0.30416032	0.30383372	0.30448691	0.08146015	0.08138108	0.08153922	0.2678198	0.2667459	0.2688980
1953	0.29378244	0.29345624	0.29410865	0.07497786	0.07490451	0.07505122	0.2552156	0.2541058	0.2563302
1954	0.28537437	0.28502508	0.28572366	0.06961119	0.06954456	0.06967782	0.2439294	0.2427071	0.2451577
1955	0.28060850	0.28030974	0.28090725	0.06711343	0.06705334	0.06717352	0.2391711	0.2381075	0.2402394
1956	0.27925313	0.27897877	0.27952749	0.06631364	0.06625452	0.06637277	0.2374679	0.2364862	0.2384537
1957	0.27736046	0.27707246	0.27764847	0.06571318	0.06564887	0.06577748	0.2369234	0.2358858	0.2379656
1958	0.27460870	0.27431863	0.27489877	0.06508104	0.06502128	0.06514081	0.2369956	0.2359403	0.2380555
1959	0.27106733	0.27079727	0.27133739	0.06412469	0.06406713	0.06418226	0.2365637	0.2355682	0.2375634
1960	0.26731104	0.26702366	0.26759841	0.06360852	0.06354600	0.06367105	0.2379570	0.2368829	0.2390359
1961	0.26785462	0.26760049	0.26810876	0.06447037	0.06441592	0.06452482	0.2406916	0.2397435	0.2416435
1962	0.26441405	0.26418432	0.26464377	0.06351425	0.06345375	0.06357475	0.2402076	0.2393386	0.2410797
1963	0.25929945	0.25904508	0.25955382	0.06240565	0.06234707	0.06246423	0.2406702	0.2396897	0.2416547
1964	0.25727696	0.25703486	0.25751907	0.06252520	0.06247172	0.06257868	0.2430268	0.2420862	0.2439711
1965	0.25511606	0.25486232	0.25536979	0.06241176	0.06235249	0.06247104	0.2446407	0.2436465	0.2456389
1966	0.25221862	0.25197804	0.25245920	0.06192001	0.06186118	0.06197884	0.2455013	0.2445476	0.2464588
1967	0.24983110	0.24959918	0.25006302	0.06151110	0.06145852	0.06156367	0.2462107	0.2452827	0.2471422
1968	0.24701287	0.24677488	0.24725086	0.06103796	0.06098657	0.06108935	0.2471044	0.2461414	0.2480712
1969	0.24284007	0.24261669	0.24306346	0.06014774	0.06009537	0.06020011	0.2476846	0.2467649	0.2486077
1970	0.23967432	0.23943715	0.23991148	0.05959760	0.05954873	0.05964647	0.2486608	0.2476719	0.2496535
1971	0.23399709	0.23379222	0.23420196	0.05758563	0.05753377	0.05763749	0.2460955	0.2452198	0.2469743
1972	0.23054209	0.23032384	0.23076035	0.05640608	0.05636151	0.05645065	0.2446672	0.2437211	0.2456169
1973	0.22580034	0.22558057	0.22602012	0.05443181	0.05438511	0.05447851	0.2410617	0.2400890	0.2420382
1974	0.21882220	0.21861651	0.21902788	0.05111168	0.05106682	0.05115654	0.2335763	0.2326370	0.2345194
1975	0.21681249	0.21660517	0.21701981	0.05043538	0.05038583	0.05048494	0.2326221	0.2316664	0.2335818
1976	0.21494543	0.21469900	0.21519185	0.04951295	0.04946995	0.04955595	0.2303513	0.2292067	0.2315015
1977	0.21402788	0.21379262	0.21426314	0.04855157	0.04850797	0.04859517	0.2268469	0.2257494	0.2279498
1978	0.21208301	0.21186684	0.21229919	0.04771696	0.04767417	0.04775975	0.2249919	0.2239738	0.2260146
1979	0.21059969	0.21037098	0.21082840	0.04723294	0.04719278	0.04727309	0.2242783	0.2231941	0.2253678
1980	0.20812675	0.20790239	0.20835111	0.04671720	0.04667140	0.04676299	0.2244651	0.2233886	0.2255469
1981	0.20325411	0.20302309	0.20348512	0.04475103	0.04471063	0.04479142	0.2201728	0.2190383	0.2213132
1982	0.20024907	0.20001032	0.20048782	0.04314627	0.04310606	0.04318648	0.2154630	0.2142733	0.2166594
1983	0.19743360	0.19719381	0.19767340	0.04221237	0.04217211	0.04225263	0.2138054	0.2125934	0.2150242

References

Jenkins, S. P. (1999). *Ineqdeco: Stata module to calculate inequality indices with decomposition by subgroup.* (Statistical software component)
